# Navigating the Spectrum of Two Pediatric COVID-19 Complications: Multi-System Inflammatory Syndrome in Children and Post-Acute Sequelae of SARS-CoV-2 Infection

**DOI:** 10.3390/jcm13041147

**Published:** 2024-02-18

**Authors:** Simon Parzen-Johnson, Ben Z Katz

**Affiliations:** 1 Section of Infectious Diseases, Biological Sciences Division, University of Chicago, 5841 South Maryland Avenue, MC 6082, Chicago, IL 60637, USA; 2 Division of Infectious Diseases, Feinberg School of Medicine, Northwestern University, 225 E Chicago Avenue, Chicago, IL 60611, USA

**Keywords:** PASC, COVID-19, pediatrics, MISC, PIM, ME/CFS

## Abstract

Purpose: This review summarizes the current scope of understanding associated with two common post-infectious complications associated with COVID-19 infection: Multi-System Inflammatory Syndrome in Children (MIS-C) and Post-Acute Sequelae of SARS-CoV-2 infection (PASC). It identifies current gaps in the knowledge and issues that may limit the ability to fill these gaps. This review provides a framework to drive continued research. Methods: A comprehensive review of the current literature was performed, identifying seminal articles describing the emergence of MIS-C and PASC, and works from the literature focused on the clinical implications and pathophysiologic understanding of these disorders. Findings: Although pediatric patients experienced few severe cases of acute COVID-19 infection, the burden of disease from post-infectious sequelae is substantial. Mortality is low, but morbidity is significant. There are still numerous unknowns about the pathophysiology of both MIS-C and PASC; however, with widespread immunity developing after increased vaccination and prior infection, it may be difficult to perform adequate prospective studies to answer pathophysiologic questions. Long-term sequalae of MIS-C seem to be minimal whereas, by definition, PASC is an ongoing problem and may be severe. Implications: The rapid sharing of information regarding novel conditions such as MIS-C and PASC are key to interventions related to future post-infectious sequelae outside of those stemming from COVID-19. Although MIS-C seems unlikely to return as a clinical condition in substantial numbers, there is still significant learning that can be gleaned from existing patients about general aspects of epidemiology, equity, and pathophysiology. There is significant morbidity associated with PASC and additional resources need to be dedicated to determining appropriate and effective therapies moving forward.

## 1. Introduction

Critical illness due to Severe Acute Respiratory Syndrome-Coronavirus-2 (SARS-CoV-2) infection is rare in children, especially in those who were previously healthy [1]. However, two post-infectious sequelae emerged during the pandemic that had significant impact on the morbidity associated with pediatric SARS-CoV-2 infections: Multi-System Inflammatory Syndrome in Children (MIS-C) and Post-Acute Sequelae of SARS-CoV-2 Infection (PASC or Long COVID). These two clinical entities are both temporally related to acute SARS-CoV-2 infection, yet they have drastically different natural histories and management strategies. In this review, we highlight their identification and what is known about their pathophysiology, their presentation, and management. With this information, we hope that future research on PASC, MIS-C, and other post-infectious sequelae will be better focused on disease identification and therapies. Although these diagnoses are associated with COVID-19, other new syndromes associated with future pandemics are likely to emerge, and the utilization of pathophysiologic knowledge and clinical outcomes from MISC and PASC will be central to our ability to address them.

## 2. Multi-System Inflammatory Syndrome in Children (MIS-C)

### 2.1. Pathophysiology and Identification

MIS-C is a uniquely severe presentation following SARS-CoV-2 infection, seen mainly in pediatric patients. It emerged as a clinical entity in early 2020, and although it has been described for over three years, the case definitions remain broad and diagnosis can be difficult. Initial reports described a syndrome of systemic inflammation resembling Kawasaki disease, with fever, variable rash, generalized extremity pain, and gastrointestinal symptoms including diarrhea, emesis, and abdominal pain about 4 weeks following acute COVID-19 infection [2]. In severe cases, patients had shock, multiple organ dysfunction, and sometimes pleural, pericardial, and ascitic effusions [2].

There are many hypotheses about the pathophysiology of systemic inflammation driving the clinical presentation of MIS-C. It is difficult to distinguish patients with severe COVID-19 infection and those with MIS-C. Shared characteristics include profound cytokine responses, decreased absolute T-cell numbers with increased activation, elevated markers of angiopathy, and increased plasmablast production [3]. When applying multi-omics to a cohort of children with severe COVID-19, MIS-C, and healthy controls, there are common HLA alleles seen in patients with MIS-C and severe COVID-19 that suggest an underlying genetic predilection to both serious conditions [4]. Beyond these findings, a recent study by Lee and colleagues performed whole-exome or whole-genome sequencing on 558 pediatric patients with MIS-C to identify potential monogenic variants that predicted the development of MIS-C over severe COVID-19 pneumonia. In their cohort, 1% of patients (significantly higher than in the general population) shared a common deficiency in genes related to OAS1, OAS2, or RNAse L. Because these genes are all involved in the innate immune response against viruses, their deficiency might explain the development of MIS-C in a few patients [5]. We posit that if other such genes can be found to be lacking in patients who develop MIS-C, that this could explain, from a host perspective, why certain pediatric patients developed MIS-C after mild COVID-19 infection while others, even those within the same household exposed to the same viral variant, were spared.

### 2.2. Diagnosis

MIS-C has many mimickers and thus posed a diagnostic challenge. While initially confused with Kawasaki disease (KD) due to overlapping signs and symptoms, it is now easier to distinguish these two diseases [6]. The median age of patients with KD and shock is <3 years, while for those with MIS-C the median age is >9 years. Significant abdominal pain, lymphopenia, myocardial dysfunction, and extremely elevated levels of N-terminal pro B-type natriuretic peptide (>10,000 pg/mL) are rare in KD but common in MIS-C [7]. Additionally, viral upper respiratory tract symptoms 2–6 weeks prior to presentation are common in MIS-C, but not in KD.

A similar distinction can be made between MIS-C and severe COVID-19, because, in contrast to acute COVID-19, children with MIS-C have few respiratory symptoms [8]. Although some children with MIS-C require mechanical ventilation, this is for cardiovascular stabilization as opposed to respiratory decompensation, as seen in acute COVID-19 [2,8].

Both the World Health Organization (WHO) and the Centers for Disease Control (CDC) created case definitions to assist with diagnosis, management, and surveillance efforts of MIS-C.

CDC Case Definition [9]:

- Patients less than 21 years of age with fever, laboratory evidence of inflammation, and clinically severe illness requiring hospitalization with multisystem organ involvement. No alternative plausible diagnosis. Current or recent SARS-CoV-2 infection within 4 weeks prior to onset of symptoms.

WHO Case Definition [10]:

- Patients less than 19 years of age with fever for three or more days, clinical signs of multisystem involvement with elevated markers of inflammation, no microbial cause of inflammation, and evidence of SARS-CoV-2 infection or exposure to COVID-19.

With the utilization of these case definitions, large case series were published highlighting the variable presenting symptoms and severity of the disease. In 2023, new case definitions of MIS-C were released which included additional clinical (cardiac, mucocutaneous, GI, etc.) and laboratory (C-Reactive Protein > 3.0 mg/dL, Platelet < 150,000 cells/μL) features, but MIS-C remains a diagnosis of exclusion. In addition to fever, gastrointestinal symptoms emerged as the most common clinical finding, followed by rash, conjunctival injection, and oropharyngeal findings [11]. Neurologic symptoms are common and range in severity from headache to life-threatening manifestations including stroke, cerebral edema, and demyelination syndromes [12].

Establishing the temporal relationship with prior SARS-CoV-2 infection was variable among case series; some patients tested positive by polymerase chain reaction for SARS-CoV-2 on presentation, others had positive serologies, and some only had exposure histories [11]. Although patients have been reported to have MIS-C without laboratory evidence of prior SARS-CoV-2 infection, these patients may very well have a different diagnosis (i.e., Kawasaki disease, systemic juvenile idiopathic arthritis, systemic lupus erythematosus, etc.). Establishing the association of preceding SARS-CoV-2 infection has been complicated by vaccination and rising infection rates in the population because of the consequent need to now distinguish vaccine immunity (manifested by SARS-CoV-2 anti-spike protein antibody) and natural immunity (manifested by nucleocapsid antibody). Acknowledging limitations due to inconsistencies with diagnosis, outcomes are reassuring, with a mortality of less than 1.5% in children [8,11], and most patients have resolution of disease without significant long-term morbidity [13].

### 2.3. Treatment, Epidemiology, and Outcomes

Treatment for MISC is focused on the treatment of systemic inflammation with medications like intravenous immunoglobulin (IVIG) and steroids. When first described, therapy was modeled on that used for KD, and IVIG was typically administered, with apparent benefit. Many clinicians noted improved clinical outcomes when corticosteroids were added to IVIG, and retrospective studies demonstrated improvements in myocardial function when dual therapy was administered compared with IVIG alone [14,15]. However, these findings are not universal [16,17]. Son et al. (2021) found that IVIG with glucocorticoids was associated with a decreased risk of starting vasopressors, an increased need for immunomodulatory therapy, and cardiovascular dysfunction when compared to IVIG alone [15]. McArdle et al. (2021) found a similar decreased risk of progression to the need for increased immunomodulatory therapy, but they found no significant difference in clinical progression (needing inotropic support or mechanical ventilation) or death when comparing IVIG and glucocorticoids to either therapy alone; however, they included patients that did not meet any of the strict case definitions of MIS-C [17]. A meta-analysis by Ouldali et al. (2023) found improved cardiovascular outcomes associated with IVIG plus glucocorticoids when compared to either medication alone. Combination therapy was also associated with the faster resolution of fever and decreased need for secondary therapies when compared to glucocorticoids alone [18]. Potential reasons for the reported differences in response to therapy in different series, besides different case definitions, include the different SARS-CoV-2 variants and the vaccination status of the children affected [19,20].

Individual centers have developed unique diagnostic and treatment protocols, although most involve IVIG and steroids. These variations illustrate the continued need for rigorous studies on the diagnosis and treatment of MIS-C [21], although this will be challenging due to continued waning case volumes [22].

Through various national reporting databases, epidemiologic studies showed that MIS-C occurred disproportionately in Black and Hispanic patients [23]. This was initially postulated to be secondary to the increased incidence of SARS-CoV-2 infections among these groups. However, as surveillance data and modeling improved it was found that when controlling for rates of SARS-CoV-2 infection, Black and Hispanic patients were still more likely to develop MIS-C [24]; whether this is due to genetic or environmental factors (or both) is still unknown.

Interestingly, there appeared to be shifting epidemiology of the disease itself over the course of the COVID-19 pandemic. Initial surveillance data linked spikes in incidence of MIS-C to spikes in SARS-CoV-2 infection, with MIS-C increases occurring approximately 4 weeks after the largest initial SARS-CoV-2 outbreaks [2]. However, as the pandemic continued, this relationship became less pronounced. In the summer and winter of 2021, increases in SARS-CoV-2 caseloads did not lead to proportioned peaks of MIS-C in the weeks following [22]. Likely, this was due to differences in the viral variant causing the acute infection; markedly fewer and less severe MIS-C cases were reported when the delta and omicron variants were the predominant circulating strain than when the alpha variant predominated [25,26]. Viral variants could differ in cellular tropism, the ability to persist, severity, or other factors that may influence the likelihood of developing MIS-C. Vaccination rates also increased during 2021, and the risk of MIS-C was lower following vaccination in children [27], although immunizations for school-aged children were not authorized in the United States until after the delta variant peaked. Hopefully, with the high levels of population immunity and vaccination, along with decreasing pathogenicity, as SARS-CoV-2 continues to evolve, severe COVID-19 epidemics will be mainly a thing of the past and MIS-C will seldom be seen.

## 3. Post-Acute Sequelae of SARS-CoV-2 Infection (PASC or Long COVID)

### 3.1. Pathophysiology and Identification

Although the acute presentation of PASC is not as severe as MISC (Table 1), its long-term impact is still evolving and research about its underpinnings and potential therapy are paramount. As early as July 2020, reports started appearing regarding long-term sequelae of acute COVID-19 infection. By September 2020, “Post-COVID-19 Condition (PCC)” became an official ICD-10 classification and has subsequently been referred to as post-acute sequelae of COVID-19 (PASC) or Long COVID. The WHO published the first case definition of PCC/PASC in December 2021 [28] and a similar definition was subsequently published for pediatric patients [29]. Both definitions include fatigue, shortness of breath, and cognitive dysfunction that must have an impact on everyday functioning. Symptoms can be new-onset following initial recovery from COVID-19 or persistent after the initial illness and may fluctuate or relapse.

WHO Case Definition:

- Patients with a history of probable or confirmed SARS-CoV-2 infection, usually 3 months from the onset of COVID-19, with symptoms that last for at least 2 months and cannot be explained by an alternative diagnosis.

Pediatric Case Definition:

- Patients with a history of confirmed SARS-CoV-2 infection who have one or more persisting physical symptoms for a minimum of 12 weeks after initial COVID-19 testing that cannot be explained by an alternative diagnosis.

Similar post-infectious syndromes have been seen following other systemic illnesses, the most common of which is myalgic encephalomyelitis/chronic fatigue syndrome (ME/CFS) following infectious mononucleosis (IM). Over 10% of patients with IM meet the criteria for ME/CFS 6 months after their bout of IM [30,31,32]. Many experts believe that PCC/PASC is the latest example of a post-infectious fatigue syndrome like ME/CFS [33].

The pathophysiology of PASC is unknown but may be due to persistent viral infection or autoimmune/post-infectious inflammatory changes [34]. Recent research has focused on alterations to metabolic pathways in patients with PASC [35], with a specific focus on the central nervous system (CNS) [36,37]. There are also numerous hypotheses based on prior experience with post-infectious sequalae from other disease processes. For example, molecular mimicry may be a driving mechanism for the long-term symptoms seen in PASC, as early in the pandemic, Wang and colleagues found that auto-antibody activity was significantly increased after infection with SARS-CoV-2 [38]. This finding, when paired with evidence of cross-reactivity between neutralizing SARS-CoV-2 antibodies and mammalian self-antigens [39], suggests that both the antibodies and the environment play a role in molecular-mimicry-driven autoimmune activity in patients following COVID-19 infection [40]. Another potential pathophysiologic mechanism focuses on perturbations of the microbiome and virome after SARS-CoV-2 infection, as has been postulated to be the case for inflammatory bowel disease, diabetes, and ME/CFS [40,41]. Immunological perturbations may also play a role [42,43]. Finally, very recent data have been published linking a reduction in serotonin levels to one or more of the above mechanisms [44].

### 3.2. Diagnosis

When the first reports of PASC appeared, the number of patients reported to have lingering symptoms following COVID-19 was as high as 60% or more [45]. However, fatigue, cognitive dysfunction and sleep disturbance are very common in the general population and could have been attributed to the psychological consequences of the pandemic itself (e.g., school closures, lockdowns, and social isolation). It was only after controlled trials were published that more realistic numbers became available.

There is significant heterogeneity amongst the published studies regarding the incidence of PASC in pediatric patients. This has made it difficult to assess the true risk of PASC development after COVID-19 infection in children. One contributor to this uncertainty is that some studies chose not to distinguish between symptoms more likely to be related to the episode of acute COVID-19 itself (e.g., myocarditis [46] and respiratory symptoms [47]) and those that are often seen with other post-viral fatigue syndromes (e.g., fatigue and cognitive dysfunction). One study clearly differentiated the onset of respiratory symptoms that dominated the first 4 weeks following initial COVID-19 infection from sleep disturbances and behavioral symptoms that dominated in the 12-to-24-week period following acute infection [48]. A second contributor to this uncertainty is that due to broad definitions and characterizations of symptoms, some studies showed alarmingly high rates of symptoms of up to 40–50%, even in uninfected controls [46,49].

One group of studies found no difference between COVID-19 cases and controls in the relative risk of developing symptoms consistent with PASC. These studies included the CLoCk study from England [48,49], data from the CDC [50,51], and data from Europe [52,53]. A second group of studies showed ≤10% difference between cases and controls and represents the largest number of reports. This group includes data from the RECOVER (REsearching COVid to Enhance Recovery) study [46], and studies from Switzerland [54], Germany [55], Denmark [20,56], England [48,57], Japan [58], and elsewhere [59]. The last group of studies yielded the highest estimates of PASC in children, of >15% [29,47,60,61]. Among the included studies, the most common symptoms of PASC were fatigue, headache, concentration difficulties, sleep disturbances, behavioral changes, and abdominal pain. Risk factors for the development of PASC included older age, severity of acute illness, comorbidities (including mental health issues at baseline) and being female [20,29,53,55,56,60]. The most comprehensive case review to date [62], including 31 studies and over 15,000 patients, found that 16% of pediatric patients had ≥1 symptom 3 months following their initial infection. This figure is on the high end based on the studies just reviewed; however, the included studies had significant heterogeneity and often included persistent respiratory symptoms, which may be a better reflection of the severity of the initial infection than PASC.

In longer-term studies of PASC, symptoms diminish with time [60,63], and in one study there were no differences in symptoms between cases and controls after 6–9 months [63]. This diminution of symptoms over time is similar to what is seen in adolescents who meet criteria for CFS following infectious mononucleosis, again supporting the notion that PASC and CFS following IM are related disease processes [32]. In one preliminary study that directly compared 19 children with PASC to 19 age- and gender-matched children with a self-reported diagnosis of ME/CFS, the children with ME/CFS had worse severity and longevity of symptoms, although symptoms in both groups declined over time [64].

### 3.3. Treatment and Outcomes

As has been summarized above, whether due to the infection itself or the conditions brought about by the pandemic (e.g., school closures and social isolation), about 15% of children and adolescents will have 3 months or more of symptoms such as fatigue, brain fog, headache, sleep disturbances, behavioral changes and abdominal pain following COVID-19. These symptoms interfere with a child’s daily activities, including school, and these children require help. At present, there is no cure for PASC. However, as symptoms tend to diminish with time there are therapies that have been shown to speed recovery for both CFS following IM and PASC, the most promising of which is cognitive behavioral therapy (CBT). The CBT modalities that have been studied for PASC include a graded exercise component, which whether alone or in combination with CBT appears to offer the best chance of speeding recovery, although studies have found that even graded exercise alone without CBT may be helpful [65,66,67]. A Cochrane review of eight randomized controlled trials in adults with ME/CFS [68] showed benefit to graded exercise, while three randomized controlled trials in adolescents with ME/CFS [69,70,71] also showed the benefit of CBT.

Long-term follow-up for PASC is not yet available since the pandemic first began at the end of 2019. However, again it may be reasonable to extrapolate findings from studies of ME/CFS. One such study on pediatric patients with ME/CFS found that ~65% of patients showed sustained recovery, after a mean follow-up 2.7 years, with online CBT alone [72]. In adults with ME/CFS, 37–70% have sustained recoveries up to 10 years later [73]. These findings are encouraging for patients with PASC. Future long-term follow-up studies focused on this important patient population are certainly warranted.

## 4. Conclusions

The COVID-19 pandemic generally spared children from severe disease. However, two post-infectious sequelae emerged. The first, MIS-C, although generally not life-threatening, was seen almost exclusively in children and had the potential to garner significant morbidity. The other, PASC, is less commonly seen in children than in adults, but has potential for significant long-term morbidity. Whether resulting from the infection itself or the challenging conditions associated with the COVID-19 pandemic, the pathophysiology of PASC is less well understood than that of MIS-C, and unfortunately its treatment remains much less satisfactory.

## 5. Future Directions

As mentioned, one of the most important future directions will be to characterize the long-term complications associated with MIS-C and PASC. There are ongoing studies looking at cardiac and neurologic recovery. Initial studies looking at follow-up echocardiography in the months following discharge have shown that most patients have full recovery without evidence of cardiac dysfunction [74]. In-depth studies utilizing cardiac MRI have had mixed results, with some showing non-specific continued signs of subclinical cardiac dysfunction, but most found significant recovery in the months after discharge [75,76]. Studies such as the National Heart Lung and Blood Institute Study on Long-term Outcomes after the Multisystem Inflammatory Syndrome In Children (MUSIC) hope to further characterize resolution over 5 years [77].

Given the similar clinical profile of patients with ME/CFS following infectious mononucleosis and PASC, an emphasis on long-term follow up was highlighted as an important facet of clinical care early on. Identifying risk factors for non-recovery and appropriate clinical management is a central goal in the care of these patients. Prospective studies are ongoing. Partnerships with appropriate medical and psychology providers should help to unravel the disease’s pathophysiology and lead to the development of new treatments and preventatives going forward. Additionally, there should be increased emphasis on improving available resources and research for pediatric patients. Studies such as the Long Haul COVID-19 Rehabilitation and Recovery Research Program (LHC Rehab), which is enrolling patients >18 years old, should have a companion study in pediatrics.

Continued research focused on understanding the biochemical and physiological underpinnings of MIS-C and PASC will undoubtedly inform us about other complex, common disease processes. For example, there are similarities between the inflammatory signature seen in MIS-C and systemic lupus erythematosus (SLE) [78]. Finally, just as we have learned much about PASC following COVID-19 from ME/CFS following mononucleosis, we hope that future studies focused on PASC will then be able to inform the field of ME/CFS as well.

## Figures and Tables

**Table 1:** Comparison of MIS-C and PASC.

	Patient Population	Onset	Signs/Symptoms	Hypothesized Pathophysiology	Treatment
MIS-C	Predominantly patients aged 7–18 years old (can rarely occur in adults)	Acute	Abdominal pain, fever, cardiac dysfunction, rash, conjunctivitis, elevated inflammatory markers	Hyperinflammatory response, autoimmune phenomenon	IVIG, Steroids
PASC	Predominantly adult patients, and within pediatrics more likely to occur in pre-teen and teenage patients	Subacute to chronic	Fatigue, shortness of breath, and cognitive dysfunction that must have an impact on everyday functioning	Persistent viral infection, autoimmune phenomenon, post-infectious inflammatory disease	Cognitive Behavioral Therapy, Graded Exercise Therapy

## Abbreviations

Severe Acute Respiratory Syndrome-Coronavirus-2 (SARS-CoV-2), Multi-System Inflammatory Syndrome in Children (MIS-C), Post-Acute Sequelae of SARS-CoV-2 infection (PASC), Kawasaki Disease (KD), World Health Organization (WHO), Centers for Disease Control (CDC), Intravenous Immunoglobulin (IVIG), Post-COVID-19 Condition (PCC), Myalgic Encephalomyelitis/Chronic Fatigue Syndrome (ME/CFS), Central Nervous System (CNS), Cognitive Behavioral Therapy (CBT), Systemic Lupus Erythematosus (SLE).
